# Compulsory Education and the Responsibility of the Psychiatrist: Instrumental Case Study

**DOI:** 10.1192/j.eurpsy.2026.11160

**Published:** 2026-07-31

**Authors:** L. Dijkhuis

**Affiliations:** Medical Student, University of Amsterdam, Amsterdam, Netherlands

## Abstract

**Introduction:**

Education has been compulsory for children since a hundred years ago. In Sweden, one-third of the students liked school very much, one-third were indifferent, and one-third disliked school very much *—* there is little reason to believe that it is different in The Netherlands (Rooy Wereldbibliotheek 2018; 226). Psychiatrists should research compulsory education as a case of compulsory care on: 1. the only way of eliminating serious harm, 2. proportionate (not excessive for the serious harm that needs to be resolved), 3. effective (yielding results). Harm might be assessed per Biesta’s purposes of education from the book Good Education in an Age of Measurement. These purposes are: qualification, socialization, and subjectification (Biesta Paradigm Publishers 2010; 5).

**Objectives:**

Identify experiments to research compulsory education as a case of compulsory care.

**Methods:**

Instrumental case study by one reviewer. The case was the task: describe your design of your dream compulsory education. The participant was a Dutch female medical student in her twenties.

**Results:**

The participant’s dream compulsory education was that it ended at the age of fourteen years old instead of eighteen:

*Primary school (NL *basisschool* ) would run until the age of fourteen years old instead of twelve. The compulsory subjects were: language, math, and drawing. The rest of the school time was reserved for free-learning. The school had a big catalogue with the filters: difficulty level (six levels like CEFR), subject, and type of Gardner’s intelligence. Furthermore, it had study guides which were as informative as Lonely Planet travel guides and the Ipad Mini was the new slate. Students could choose between classroom learning (i.e. active teacher) and homeworkguidance learning (i.e. passive teacher). Tests were as flexible as the Cambridge English Test.

*Secondary school (NL *middelbare school* ) would run like the preparatory course of the Dutch Royal Conservatoire (i.e. by invitation only when it has become clear during the audition for a tertiary school that there is reason to do so).

**Conclusions:**

The result continues the old trend of viewing children as unfinished. Since the eighteenth century this trend has changed to seeing children as untouched in a nostalgic romantic sense (Rooy Wereldbibliotheek 2018; 273-276). Furthermore, the result changes the trend of compulsing adolescents. Puberty was one of the reasons to extend the years of compulsory education (Rooy Wereldbibliotheek 2018; 137). Australian aboriginals do not compulse male adolescents: a walkabout ceremony at the age of twelve years old or thirteen. The main identified experiments to research compulsory education as a case of compulsory care are focused on Biesta’s qualification and - subjectification: an experiment on whether to view the child as unfinished or as untouched and an experiment on how the child scores oneself in Virk’s model Four quadrants of passion.

**Disclosure of Interest:**

None Declared

